# A Proving of Glonoine, Crude

**Published:** 1899-10

**Authors:** 


### A PROVING OF GLONOINE, CRUDE.

We had the distinguished honor of being blown up with Nitro-glycerine a few nights since. But reserve your obituaries, brethren and friends. Funeral postponed. We had business down in the city, and returning before midnight boarded a street-car. After reaching one of the most aristocratic sections of our city, the book we were reading suddenly flew out of our hand, and we as suddenly after it. We were seated directly over the wheel which exploded the n. g., which had been carelessly left on the track by a few infamous scoundrels. The detonation was terrific, and after we had recovered from the shock and extricated ourselves from the upheaved and up-torn flooring and blinding smoke, we could not hear a sound for some minutes. But this wore off. The car wheel was blown into "flinders." A noticeable thing was that the glass in the window against which our back rested, which was directly over the wheel, broke into innumerable fragments, but almost wholly in square or rectangular pieces: very few sharp splinters. We prefer to take our Glonoine in the highly attenuated form, as we now fully realize that a proving of the crude chemical is attended with no marked mental or other symptoms, except those of an indecent haste to go elsewhere.—*American Homœopathist*, August 15th, 1899.

[Dr. Kraft now knows how Lazarus felt when he "came forth."—Ed.]

